# Identification of Levothyroxine Antichagasic Activity through Computer-Aided Drug Repurposing

**DOI:** 10.1155/2014/279618

**Published:** 2014-01-30

**Authors:** Carolina L. Bellera, Darío E. Balcazar, Lucas Alberca, Carlos A. Labriola, Alan Talevi, Carolina Carrillo

**Affiliations:** ^1^Medicinal Chemistry, Department of Biological Sciences, Faculty of Exact Sciences, National University of La Plata, 47 y 115, La Plata (B1900AJI) Buenos Aires, Argentina; ^2^Instituto de Ciencia y Tecnología Dr. César Milstein (ICT Milstein), Argentinean National Council of Scientific and Technical Research (CONICET), Saladillo 2468, Ciudad Autónoma de Buenos Aires (C1440FFX), Argentina; ^3^Instituto de Investigaciones Bioquímicas de Buenos Aires, Argentinean National Council of Scientific and Technical Research (CONICET), Avenida Patricias Argentinas 435, Ciudad Autónoma de Buenos Aires (C1405BWE), Argentina

## Abstract

Cruzipain (Cz) is the major cysteine protease of the protozoan *Trypanosoma cruzi*, etiological agent of Chagas disease. A conformation-independent classifier capable of identifying Cz inhibitors was derived from a 163-compound dataset and later applied in a virtual screening campaign on the DrugBank database, which compiles FDA-approved and investigational drugs. 54 approved drugs were selected as candidates, 3 of which were acquired and tested on Cz and *T. cruzi* epimastigotes proliferation. Among them, levothyroxine, traditionally used in hormone replacement therapy in patients with hypothyroidism, showed dose-dependent inhibition of Cz and antiproliferative activity on the parasite.

## 1. Introduction

Chagas disease is a tropical parasitic disease caused by the flagellate protozoan *Trypanosoma cruzi*. *T. cruzi* life-cycle includes both vertebrate and invertebrate hosts. 80 to 90% of infections in humans occur when haematophagous triatomine bug feces come into contact with wounded skin or mucosae [1]. Other infection ways include blood-transfusion and congenital transmission. Even though a series of control campaigns developed by World Health Organization (WHO), Pan American Health Organization (PAHO), and national authorities have considerably reduced Chagas disease incidence in the last fifteen years, there are still almost 8 million infected people and 28 million people at risk [2–4].

**Figure 1:**
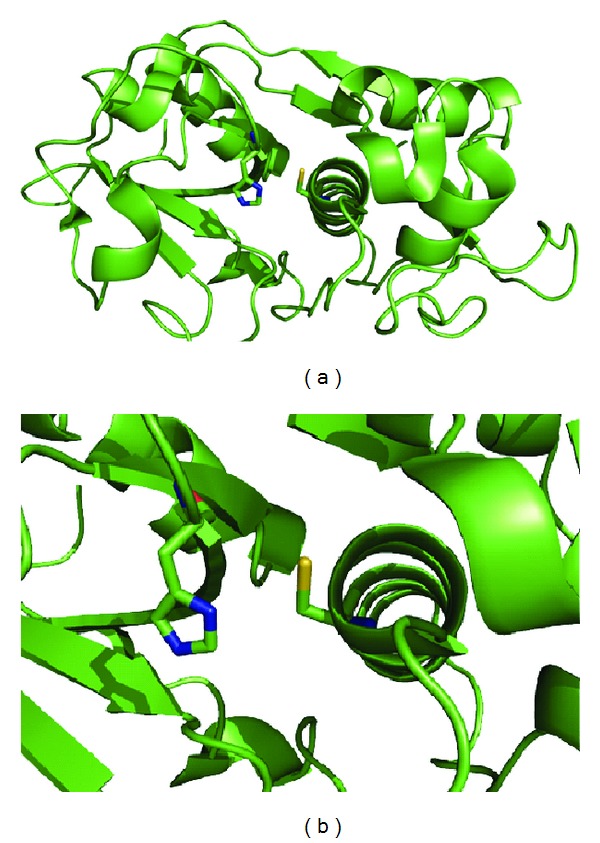
(a) Model of the cruzipain. (b) Active site of cruzipain.

Current treatment against Chagas relies on only two drugs developed during 1960s–1970s, namely, nifurtimox and benznidazole, which are not effective in the late chronic phase of the disease and present severe side effects and resistance issues [5–7]. It is worth noting, however, that important advances have been made in the fields of biochemistry and molecular biology of *T. cruzi* and novel antichagasic therapeutics [4, 8–11]. Cysteine protease inhibitors are among the most investigated candidates against *T. cruzi *[11]. Cruzipain (Cz), the major cysteine protease of the parasite, has been particularly explored as new drug target (a model of the enzyme is presented in Figure 1). This enzyme has proven to be essential for replication of the intracellular form of *T. cruzi* and plays a role in host-parasite interactions [12]. It is believed that Cz inhibition produces accumulation of the inactive precursor of the proteinase within the Golgi complex, which eventually leads to osmotic shock and cell death [13].

Virtual screening encompasses the application of a diversity of computational methods (models or algorithms) to chemical libraries or databases, in order to prioritize which of the library compounds will be sent to experimental (*in vitro *or *in vivo*) testing. Here, we present a 2D classification model derived from a 163-compound dataset of Cz inhibitors and noninhibitors. The model was later applied in a virtual screening (VS) campaign to explore the small molecule database DrugBank in order to identify novel Cz reversible inhibitors. DrugBank compiles FDA-approved and experimental drugs [14, 15], being particularly helpful to conduct VS campaigns aimed to drug repurposing (i.e., searching new therapeutic indications for already known drugs). Traditionally, second medical uses emerged from intelligent exploitation of approved or investigational drugs side effects (e.g., to exploit the aspirin antiplatelet effect to prevent heart attacks and strokes or the use of sildenafil to treat erectile dysfunction). Lately, however, knowledge-based, rational drug repositioning (chemoinformatics- and bioinformatics-based and others) has gained attention [16–19] and is being increasingly used to aid in discovering novel treatments for rare, neglected, and poverty-related conditions [20–22].

## 2. Materials and Methods

### 2.1. Dataset Compilation and Splitting

**Figure 2:**
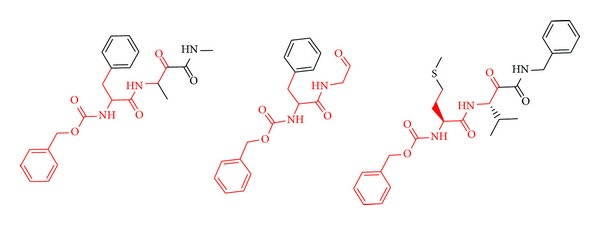
An example of MCS. The MCS of the three structures is depicted in red.

Building a computational model capable of discriminating active from inactive chemical compounds involves three fundamental steps. First, a dataset of chemical compounds whose class (active or inactive) has been experimentally determined should be compiled. Second, the dataset must be partitioned into a training set that will be used to infer (or train or calibrate) the model and an independent (hold-out) test set that will be used to assess the model predictive ability. Ideally, the training set should present an adequate balance of the active and inactive classes so that the inferred model is not biased. In our case, a 163-compound balanced dataset including 82 Cz reversible inhibitors and 81 noninhibitors was compiled from literature [23–34]. The dataset is available as Supplementary Material available online at http://dx.doi.org/10.1155/2014/279618. A relevant issue that should be addressed is how to split the dataset into representative training and test sets. It has been demonstrated that random partition is an adequate approach whenever training and test sets of similar size are selected, but more rational sampling approaches provide better results when test sets are small compared to the correspondent training sets [35, 36]. Following the latter criteria, the LibraryMCS v0.7 (ChemAxon) hierarchical clustering approach was applied in combination with the *k*-means clustering implemented in Statistica 10 Cluster Analysis module (Statsoft Inc, 2011). The fundamental idea is to identify, within the diverse dataset, groups of common chemical features to guide the selection of adequate training and test sets. LibraryMCS relies on similarity-guided maximum common substructure (MCS) to cluster a set of chemical structures without exhaustive pairwise comparison. Covalently bonded atoms are regarded as a mathematical “graph” where an atom and a bond correspond to a vertex and an edge, respectively. A common substructure is defined as a substructure present in two molecules with the same atom types and bond connections. The MCS is defined as the common substructure with the largest number of atoms or bonds (see Figure 2, e.g.) [37]. Library MCS builds the similarity matrix for the input structures and finds the MCS for the two most similar molecules. This MCS is then used to create and populate a cluster through substructure search. Such procedure is repeated iteratively until no pair of structures with similarity above a similarity threshold is found, in which case singletons are generated. Similarity-guided MCS search is used to find MCS of multiple structures efficiently, without exhaustive pairwise comparison. Thus, LibraryMCS leads to reproducible but approximate solutions [38]. Since the number of clusters in *k*-means clustering analysis is a user-defined parameter, hierarchical clustering has been applied here to define an initial partition of “*n*” objects into “*g*” groups, as suggested by Everitt et al. [39], and the groups of compounds were later optimized by *k*-means algorithm, minimizing the Euclidean distance to the group centers. A smallest common substructure of at least 9 atoms was used, and a randomly selected member from the clusters defined by the hierarchical approach was used as seed in the k-means clustering procedure. A series of descriptors computed with Dragon 6.0 (Milano Chemometrics, 2012) representing different aspects of molecular structure (viz., molecular weight, log *P*, polar surface area, number of H bonds acceptors, information index of atomic content, sum of atomic van der Waals volumes) were normalized and applied to calculate Euclidean distance. Once the clusters were separately identified in the inhibitors and noninhibitors categories, 25% of each cluster was assigned to an independent test set for validation purposes, while the remaining 75% of the clusters were retained as training set for modeling purposes. The structures of both training and test set compounds are provided as Supplementary Information.

### 2.2. Descriptor Calculation and Modeling

Molecular descriptors are numerical variables that reflect different aspects of molecular structure; as such, they are useful to derive quantitative structure-activity relationships. A total of 3764 0D–2D molecular descriptors were computed with Dragon 6.0 Academic version (Milano Chemometrics. 2010). Since the values of such descriptors are conformation independent, they are particularly suitable for their application in VS campaigns, requiring no preprocessing (e.g., conformational analysis or optimization) of the screened database structures. From the 3764 descriptors, 25 random subsets of no more than 254 descriptors were generated, and these subsets were used as descriptor pools for modeling purposes (random subset approach).

Linear Discriminant Analysis (LDA) was then conducted in order to derive a classification model capable of distinguishing Cz inhibitors from noninhibitors. LDA is a qualitative supervised learning method aimed to finding a linear combination of independent variables to differentiate between two or more categories of objects. Each object class is associated with a given value (an integer value) of an arbitrary variable that serves as class label. In our case, only two object classes (ACTIVE-Cz inhibitors and INACTIVE-noninhibitors) were considered; thus the class label assumes two observed values (1 and −1, resp.). Since the output of the function being searched is not a continuous variable but only an object category, LDA and other classificatory techniques may be useful to handle noisy data, for example, if a given experimental endpoint is associated with large variability or if experimental data from a diversity of laboratories are compiled [40].

The Discriminant Analysis module of Statistica 10 was used to build the models. A tolerance value of 0.5 was selected in order to exclude highly correlated descriptors from the model. All the coefficients linked to the models descriptors were significant at a 0.05 level. A minimum ratio of 15 between the number of training set compounds and the number of independent variables was used in order to reduce the chances of overfitting. Overfitting refers to gaining explanatory power on the training set compounds at the cost of losing predictive ability. Parsimony principle, Wilks' lambda, and the performance of the model on the independent test set were used to select the best model. Standard validation approaches (stratified leave-group out cross-validation, randomization test, and external validation) were used to assess the model's robustness and predictive ability [41]. Stratified 20-fold cross-validation and 30 randomization tests were applied. Cross-validation implies removing training set compounds, regenerating the model and using this new model to predict the removed cases. Randomization implies scrambling the dependent variable among the training set compounds (thus abolishing the structure-property relationship) and obtaining a new, “randomized” model, which should theoretically perform poorly compared to the actual model.

### 2.3. Simulated Virtual Screening Campaign

An issue that emerges from using a reduced dataset (such as our 42-compound test set) to assess the performance of ranking methods in virtual screening is that the metrics used to such purpose exhibit a higher variance compared to significantly large datasets. Experiments conducted by Truchon and Bayly [42] show that the standard deviations associated with several frequently used metrics (among them the ROCAUC) are higher for small datasets and converge to a constant value when the size of the dataset increases.

Another problem is related to the high ratio of inactives which mainly hinders the early recognition ability in what is known as the “saturation effect.” That is, for datasets with a high ratio of hits (in our case, Cz inhibitors), once hit compounds saturate the early part of the ordered list, the enrichment metric cannot get any higher. To estimate in a more realistic way the utility of our model in a real virtual screening approach, we have dispersed our test set among 444 putative noninhibitors acting as decoys. Such putative noninhibitors are highly similar compounds (0.95 similarity or more) to the test set noninhibitors and have been retrieved from PubChem. This simulated database thus contains 21 known Cz-inhibitors among 465 known or putative noninhibitors; that is, the Cz-inhibitors ratio is less than 0.05, representing a more challenging set to assess the enrichment ability of our models. Note that some of these putative noninhibitors (decoys) might actually be Cz-inhibitors; thus, the true performance of our models may be even higher than the one obtained through this simulated experiment.

### 2.4. Virtual Screening

DrugBank 3.0, a chemical database which compiles FDA approved and investigational drugs, was screened. Only approved and experimental small molecules and nutraceuticals (6684 total compounds) were considered (biotech drugs were excluded a priori). Pharmacological Distribution Diagrams (PDD) and separate Receiver Operating Characteristic (ROC) curves were constructed for both the training and test sets, in order to select the discrimant function score threshold value determining and adequate sensitivity/specificity ration, to be used in the VS campaign [43, 44]. ROC curves provide graphical insight into the specific-sensitivity balance for different model score thresholds, allowing selection of an appropriate threshold on the basis of context-dependent criteria. To build ROC curves MedCalc ROC curves analysis tool was applied (MedCalc software, 2012).

### 2.5. Inhibitory Effect on Cz Activity Assay

To study the effect of the selected compounds on Cz activity, the enzyme was partially purified by ammonium sulfate precipitation followed by affinity column chromatography on concanavalin A-sepharose (Sigma), as previously described [45]. The activity of the partially purified Cz was assayed with 250 *μ*M Bz-Pro-Phe-Arg-pNA (Sigma) as substrate, incubated in a buffer of 6, 5 *μ*M dithiothreitol (DTT) and 50 mM Tris-HCl pH 7 [46], in presence or absence of diverse compounds. The reaction was measured spectrophotometrically at room temperature at 410 nm for 5 min (Beckman CoulterTM DU530 Life Science UV-vis spectrophotometer). The values obtained were converted into pmol of hydrolyzed substrate per min by using the extinction coefficient 8.800 M^−1^ cm^−1^ (p-nitroanilines). The inhibitory effect of the selected candidates was expressed as a percentage of residual activity of Cz with respect to the assay without inhibitors.

### 2.6. Inhibitory Effects on *T. cruzi* Epimastigote Proliferation

Epimastigotes of the *T. cruzi* strain Y were cultured at 28°C in BHT medium with 20 mg/L Haemin, 20% heat-inactivated fetal calf serum and antibiotics (100 *μ*g/mL streptomycin and 100 U/mL penicillin) [47] adding the indicated levothyroxine concentration (0–200 *μ*M). Cultures were initiated at 10^6^ cells/mL, and the proliferation was followed daily by cell counting in a hemocytometer chamber. In order to follow the effect of the drug on proliferation for long periods, once the cultures reached the stationary phase (each 7 days), they were restarted by dilution at 10^7^ cells/mL with fresh medium plus levothyroxine.

## 3. Results and Discussion

Clustering procedure revealed 5 groups of at least 6 compounds in the ACTIVE category and 7 groups of at least 7 compounds in the INACTIVE class. According to MCS clustering, there are 4 compounds in the ACTIVE class which can be considered outliers (singletons or groups of only two compounds, meaning they share no MCS above the specified number of atoms with other molecules from the set), whereas the INACTIVE category presents 14 outliers. On the basis of the clustering procedure, 25% of each cluster was assigned to the test set for external validation purposes, while the remaining 75% of each cluster was assigned to the training set upon which the model was derived.

The following model was obtained through LDA:

(1) Class=0.819+0.536∗VE1_X−1.187∗C-018   +0.495∗F-048−0.688∗NsssN−0.187∗H-051 −0.427∗O-056−0.09∗Sds−5.311∗SpPosA_A,(N=121,  λ=  0.445,  F(8,112)=17.476,  p<0.00001),

where VE1_X represents the sum of the coefficients of the eigenvector associated with the last (largest negative) eigenvalue of the *chi* matrix. Such matrix is a modified adjacency matrix, obtained by weighting each bond between pairs of vertices by the edge connectivity [48]. The elements of the *chi* matrix are define as follows:

(2) $$\begin{array}{c} X_{i,j}=\{\begin{array}{cc} \frac{1}{\sqrt{m\times n}} & \text{if}\;i,j\;\text{are adjacent}\\ 0 & \text{otherwise,} \end{array} \end{array}$$

where *m* and *n* are the valences of the vertices involved. C-018 is the number of =CHX (= representing a double bound and X being a heteroatom), F-084 denotes the number of atoms F attached to carbons SP2, NsssN represents the number of tertiary nitrogens, H-051 is the number of H atoms attached to an alpha carbon, O-056 denotes the number of groups OH, Sds symbolizes the sum of the E-states of the =S atom type (thus providing information about the electron accessibility and the count of the number of atoms of such atom-type) and SpPosA_A is the normalized spectral positive sum from adjacency matrix. The magnitudes of the beta coefficients of such descriptors are, respectively, 0.564, 0.185, 0.168, 0.160, 0.137, 0.135, 0.142, and 0.108, showing that VE1_X is the most relevant independent variable of the model. It should be highlighted that the model presents a good cases per predictor ratio (around 15) which indicates a low chance of overfitting, as confirmed later in the external validation results. When using 0 as a score threshold to differentiate active from inactive compounds, the model presents 87% of good classifications among the training set inactive compounds, 90% of good classifications among the training set active compounds, and an overall of 88% good classifications. Regarding the test set, the model accurately classifies 81% of the active and 90.5% of the inactive compounds, with an overall good classification of 86%. These results seem to confirm that no overfitting has occurred, since the performance on the test set is very similar to the performance on the training set. The average performance of the randomized models was 68.4 (*sd*⁡ = 4.1) showing that the randomized models were significantly outperformed by the actual model, as expected. Cross-validation resulted in an average percentage of good-classifications of 79% (average of the result of the 20 folds); remarkably, among the worst-classified folds we found 5 of the outliers detected by the hierarchical clustering procedure.

**Figure 3:**
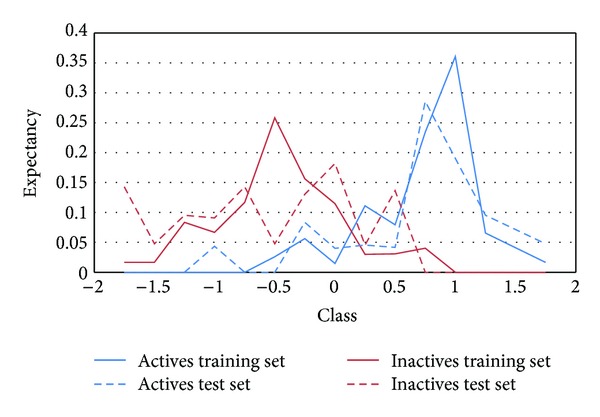
PDD showing the distribution of training and test set active and inactive compounds along the score values of the model. An acceptable superposition between both sets can be observed.

**Figure 4:**
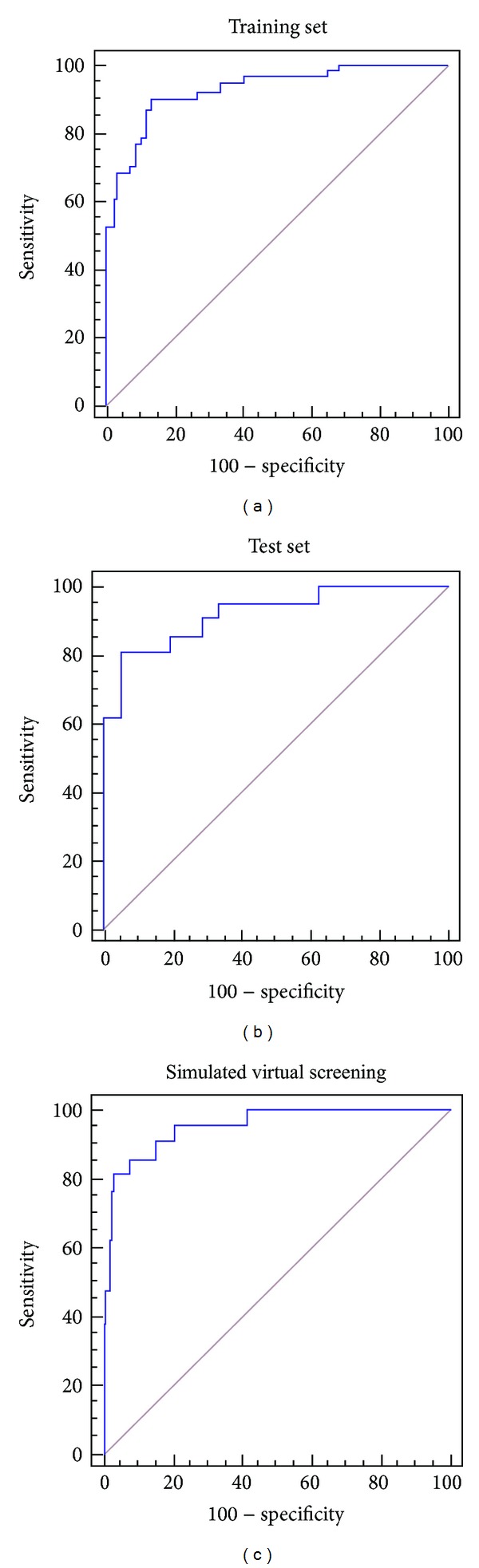
Training set (a), test set (b), and simulated database (c) ROC curves.

We resorted to PDD and ROC curves in order to optimize the chosen threshold score on a rational basis [40, 41]. Figures 3 and 4 present, respectively, the PDDs of the training and test sets, the ROC curves for the training set, test set, and the 486-compound simulated database. The area under the curve (AUC) for the training and test sets ROC curves was, respectively, 0.930 and 0.923 (1 represents perfect classification, while 0.5 represents random classification). 0.06 was selected as the cutoff value to differentiate active from inactive compounds in the VS campaign. According to the ROC curves data, this value corresponds to a sensitivity of 87% and a specificity of 88% in the training set, and a sensitivity of 81% and a specificity of 95% in the test set. As stated by Triballeau in the original application of ROC curves to VS [41], the selection of a given balance between sensitivity and specificity is not a statistical matter but a context-dependent decision. In our case, due to a limited budget to acquire and test compounds, we have prioritized specificity (i.e., reducing the chance of false positives) over sensitivity. This means that, in order to increase the chance of having positive results in the biological tests, we risk losing potentially valuable structural motifs.

Remarkably, our simulated VS campaign resulted in a ROC AUC of 0.953 (larger, in fact, that the ROC AUCs obtained for the training and test sets). Furthermore, 16 out of 21 Cz inhibitors in the dataset (76% of the total number of inhibitors) appear among the 5% best ranked compounds from the simulated database.

**Figure 5:**
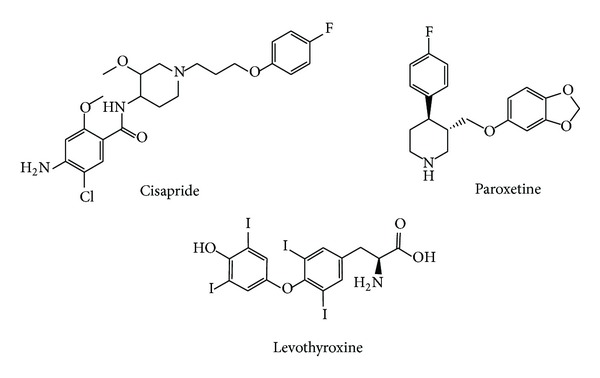
Molecular structures of the three candidates selected for enzymatic testing.

From 6684 small approved and investigational molecules of the DrugBank 3.0 database, 64 candidates were selected, with a score above the selected threshold; 54 of them correspond to approved drugs, which are the straightforward candidates for repositioning purposes. On the basis of their accessibility, 3 of them (Figure 5) were acquired and experimentally tested in the enzymatic assay on Cz. The acquired candidates were cisapride (gastroprokinetic agent, increases motility in the gastrointestinal tract), paroxetine (antidepressant, a selective inhibitor of serotonin reuptake), and levothyroxine (used in hormone replacement therapy in patients with hypothyroidism). Among them, only paroxetine had been previously tested on cruzipain (with negative results) through a quantitative high-throughput screening approach which assessed the effect of more than 197,000 candidates on the hydrolysis of the fluorogenic substrate Z-Phe-Arg-AMC [49].

**Figure 6:**
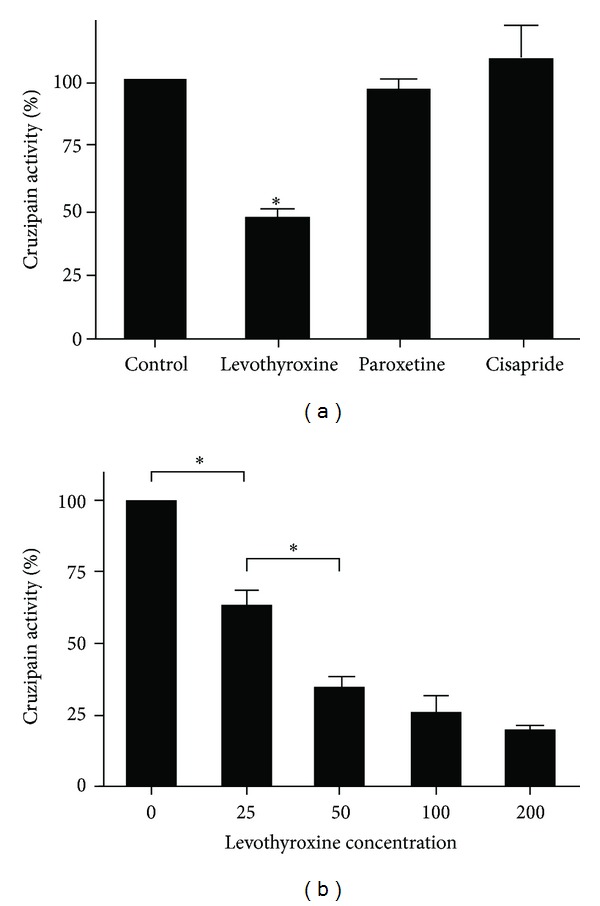
(a) Inhibitory effect of the three selected candidates on purified Cz activity. The final concentration of each compound was 50 *μ*M. Protease activity is expressed as percentage of the control condition (2% DMSO). (b) Dose-dependent inhibitory effect of levothyroxine on purified Cz activity. Levothyroxine was assayed in a concentration range of 0–200 *μ*M. Remanent Cz activity was expressed as a percentage respect to the control (0 *μ*M levothyroxine, 2% DMSO). Asterisks indicate significant differences (*P < 0.05).

Using Bz-Pro-Phe-Arg-pNA as chromogenic substrate, levothyroxine showed a significant inhibitory effect on *T. cruzi* Cz activity (Figure 6(a)). Such inhibition proved to be dose dependent on purified Cz, with an IC50 of 38.43 ± 6.82 *μ*M (Figure 6(b)).

**Figure 7:**
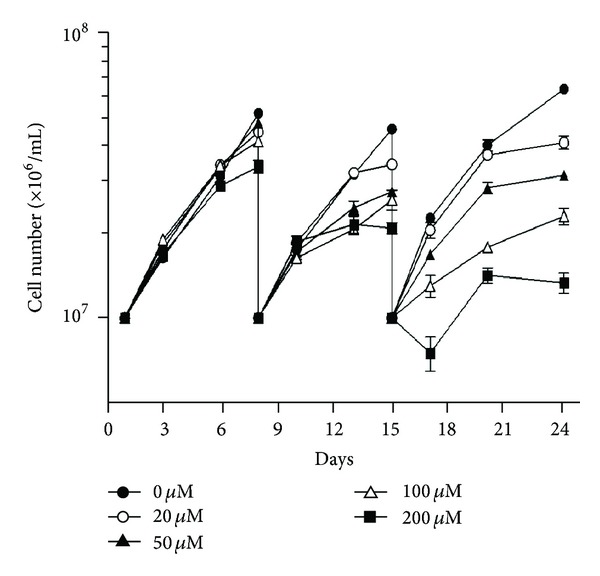
Effect of levothyroxine on *T. cruzi* epimastigotes proliferation. To determine the growth rate, 10^7^ cells/mL were seeded in BHT medium and cultured at 28°C with the indicated concentrations of the drug; the control condition was done with 0 *μ*M levothyroxine, 2% DMSO. Parasites were counted daily using a hemocytometer chamber and, once they reached the stationary phase, were rediluted at 10^7^ cells/mL with fresh medium plus the indicated levothyroxine concentration. Results represent the mean ± SD of a representative experiment.

*T. cruzi* epimastigotes proliferation was affected by levothyroxine progressively in time and in a dose-dependent manner (Figure 7). The effect was clearly notorious on the third week of assay showing a median inhibitory dose (ID_50_) of 121.76 ± 11.39 *μ*M at the middle log phase of controls (17th day).

## 4. Conclusions

An 8-descriptor conformation-independent classification model was derived from a 163-compound dataset which compiled Cz inhibitors and noninhibitors extracted from the literature. The model presented an excellent case to descriptor ratio and similar performance on both the training and the test sets which suggest good predictive ability and absence of overfitting. Since only conformation-independent descriptors were included in the model, it is particularly suitable for efficient exploration of drug libraries through VS campaigns without requiring any preprocessing of the library structures.

Having in mind the potential of knowledge-based drug repositioning to develop novel therapies for neglected and rare diseases, the model was applied in a VS campaign to select potential antichagasic drugs from the DrugBank database, which compiles approved and investigational active ingredients. PDD and ROC curve analysis were conducted in order to select a score cutoff value to differentiate active and inactive agents on a rational basis.

Three candidates were acquired and experimentally tested in enzymatic and inhibitory assays. Among them, levothyroxine (traditionally used in hormone replacement therapy in patients with hypothyroidism) showed a dose-dependent inhibition on Cz activity with concomitant effects on *T. cruzi *proliferation. The results exemplify the potential of computer-aided drug repositioning in the search of novel medications for poverty-related diseases.

## Supplementary Material

Chemical structure of the 163 compounds that compose the dataset that was used to train and validate the reported model.

## Abbreviations

LDA: Linear discriminant analysis
MCS: Maximum common substructure
ROC: Receiving operating characteristic
VS: Virtual screening.
